# Value and mechanisms of EEG reactivity in the prognosis of patients with impaired consciousness: a systematic review

**DOI:** 10.1186/s13054-018-2104-z

**Published:** 2018-08-02

**Authors:** Eric Azabou, Vincent Navarro, Nathalie Kubis, Martine Gavaret, Nicholas Heming, Alain Cariou, Djillali Annane, Fréderic Lofaso, Lionel Naccache, Tarek Sharshar

**Affiliations:** 1 Department of Physiology and Department of Critical Care Medicine, Raymond Poincaré Hospital, Assistance Publique – Hôpitaux de Paris (AP-HP), Inserm UMR 1173 Infection and Inflammation, University of Versailles Saint Quentin (UVSQ), University Paris-Saclay, Garches, Paris, France; 2 Department of Clinical Neurophysiology, Pitié-Salpêtrière Hospital, AP-HP, Inserm UMRS 1127, CNRS UMR 7225, Sorbonne Universities, Université Pierre et Marie Curie – UPMC Université Paris 06, Paris, France; 3 Department of Clinical Physiology, Lariboisière Hospital, AP-HP, Inserm U965, University of Paris Diderot, Sorbonne Paris Cité, Paris, France; 4 Department of Clinical Neurophysiology, Sainte-Anne Hospital, Inserm U894, University Paris-Descartes, Paris, France; 5 Medical ICU, Cochin Hospital, AP-HP, Paris Cardiovascular Research Center, INSERM U970, Université Paris Descartes Sorbonne Paris Cité, Paris, France; 6 Department of Neuro-Intensive Care Medicine, Sainte-Anne Hospital, Paris-Descartes University, Paris, France; 7 Clinical Neurophysiology Unit, Raymond Poincaré Hospital - Assistance – Publique Hôpitaux de Paris, INSERM U1173, University of Versailles-Saint Quentin (UVSQ), 104 Boulevard Raymond Poincaré, Garches, 92380 Paris, France

**Keywords:** Intensive care unit, Mortality, Prognosis, EEG reactivity, Spinothalamic tract, Lateral lemniscus, Brain dysfunction, Coma

## Abstract

**Background:**

Electroencephalography (EEG) is a well-established tool for assessing brain function that is available at the bedside in the intensive care unit (ICU). This review aims to discuss the relevance of electroencephalographic reactivity (EEG-R) in patients with impaired consciousness and to describe the neurophysiological mechanisms involved.

**Methods:**

We conducted a systematic search of the term “EEG reactivity and coma” using the PubMed database. The search encompassed articles published from inception to March 2018 and produced 202 articles, of which 42 were deemed relevant, assessing the importance of EEG-R in relationship to outcomes in patients with impaired consciousness, and were therefore included in this review.

**Results:**

Although definitions, characteristics and methods used to assess EEG-R are heterogeneous, several studies underline that a lack of EEG-R is associated with mortality and unfavorable outcome in patients with impaired consciousness. However, preserved EEG-R is linked to better odds of survival. Exploring EEG-R to nociceptive, auditory, and visual stimuli enables a noninvasive trimodal functional assessment of peripheral and central sensory ascending pathways that project to the brainstem, the thalamus and the cerebral cortex. A lack of EEG-R in patients with impaired consciousness may result from altered modulation of thalamocortical loop activity by afferent sensory input due to neural impairment. Assessing EEG-R is a valuable tool for the diagnosis and outcome prediction of severe brain dysfunction in critically ill patients.

**Conclusions:**

This review emphasizes that whatever the etiology, patients with impaired consciousness featuring a reactive electroencephalogram are more likely to have a favorable outcome, whereas those with a nonreactive electroencephalogram are prone to having an unfavorable outcome. EEG-R is therefore a valuable prognostic parameter and warrants a rigorous assessment. However, current assessment methods are heterogeneous, and no consensus exists. Standardization of stimulation and interpretation methods is needed.

## Background

**Fig. 1:**
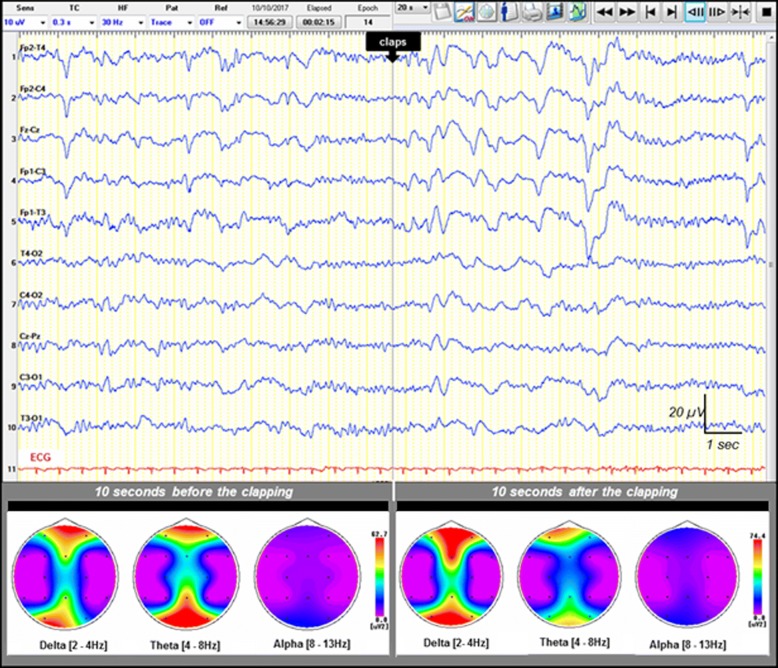
Example of a reactive electroencephalogram (EEG) following auditory stimulation (claps) of a patient with impaired consciousness. Upper: A 20-second epoch EEG sample showing a diffuse and synchronous slowing of the EEG background activity, appearing immediately after the auditory stimulus (claps) in an ICU patient with sepsis-associated encephalopathy. Recording: 20 mm/s, sensitivity: 10 μV/mm; filter settings: 0.5–70 Hz. Lower: EEG spectral power featuring topographic mapping of power of each main EEG frequency band (delta, theta, and alpha) computed 10 seconds before and 10 seconds after the auditory stimulus onset (claps). EEG changes from a theta-dominant frequency (before stimulation) into a delta-dominant one (after stimulation). Higher-power values are shown in warm colors, and cool colors depict lower power

Electroencephalography (EEG) is a clinical neurophysiology tool used to evaluate cerebral cortex activity that possesses demonstrated efficacy for the diagnosis, monitoring, and prognosis of brain disorders in critically ill patients [1–4]. Guidelines of the International Federation of Clinical Neurophysiology and the American Society of Clinical Neurophysiology provide standardized methods for EEG recording and analysis in intensive care unit (ICU) patients [1, 5–7]. EEG analysis relies mainly on the analysis of basic parameters such as the dominant frequency of background activity and its continuity, reactivity to stimuli, and the symmetry and occurrence of paroxysmal activities [1, 2, 8–11]. Many abnormal EEG patterns predict a poor outcome in critically ill patients [11–23]. Several EEG scores have been described [2, 4, 22, 24–26]. Several studies point out that electroencephalographic reactivity (EEG-R) or the absence thereof was particularly useful for prognostication in patients with impaired consciousness [8, 27–30]. Although there is no consensus regarding the definition or the methods to use in assessing EEG-R, EEG-R could be defined as diffuse and transient changes in scalp recorded EEG activity in response to sensorial external stimuli. Such stimuli may be auditory (clapping and loudly calling the patient’s name), nociceptive (pinching of limbs or nipples, compression of the fingernails or of the periosteal surfaces of bones) [31], or visual (spontaneous or forced eye opening, intermittent photic stimulation) [29, 31–39]. The amplitude and/or frequency of EEG activity may change in response to external stimulation (Fig. 1). However, EEGs merely exhibiting stimuli-induced rhythmic, periodic, or ictal discharges [36] or muscle activity or eye blink artifacts are not considered as reactive by many authors [1, 5–7]. Because visual analysis of reactivity is prone to subjectivity [40–42], automated quantitative approaches have been proposed [37]. EEG-R to nociceptive, auditory, and/or photic stimulation requires the functional integrity of peripheral sensory pathways, the brainstem, subcortical structures, and the cerebral cortex. Absent EEG-R could therefore result from a severe dysfunction of any of these structures, precluding the cortical activation by the afferent somatosensory stimuli [43]. The importance of EEG-R in predicting patient outcome in postanoxic coma has been documented in many studies since the 1960s [14, 41, 44–46]. Lack of EEG-R has been shown to be of prognostic value in postanoxic, posttraumatic, or hepatic encephalopathies [3, 8, 16, 27–29, 47]. The present review highlights and discusses the mechanisms and particular usefulness of EEG-R for determining the prognosis of patients with impaired consciousness.

## Methods

We systematically searched the literature in the PubMed database for published reports pertaining to the use of EEG-R in outcome prediction in patients with impaired consciousness, from inception until March 2018, using the following search terms: (EEG reactivity OR electroencephalogram reactivity OR reactive EEG) AND (coma OR anoxic OR cerebral anoxia OR hypoxia OR post anoxic coma OR resuscitation OR cardiac arrest OR traumatic brain injury OR TBI OR encephalopathy OR unconscious OR vegetative state OR unresponsive wakefulness syndrome OR minimally conscious state) AND (outcome OR prognosis OR prognostication OR prediction OR predictive value OR mortality OR survival OR awakening). The search yielded 202 articles. Of these, we excluded non-English-language articles (*n* = 25) as well as those for which no full text was available (*n* = 28). Of the 149 remaining articles, we included 80 publications covering assessment of EEG-R and its impact on the prognosis of patients with impaired consciousness. Among these 80 publications were 17 review articles, 2 systematic reviews [32, 48], and 61 clinical investigation papers. We then carefully read and scrutinized all of these latter 61 articles.

## Results

Data on the prognostic value of EEG-R in patients with impaired consciousness were explicitly reported in only 42 of the papers [8, 28, 30, 33, 37, 38, 44, 49–83] (*see* Table 1). Most studies in the present review assessed EEG within the first week following admission to the ICU or rehabilitation unit for postacute disorders of consciousness. EEG-R to external stimulation has emerged as an important predictor of improved outcome in a wide variety of clinical conditions [3, 8, 16, 27–29, 47], including traumatic brain injury (TBI) and anoxic brain injury [14, 16, 18, 72, 84]. Logi et al. [14] assessed the value of EEG-R in predicting consciousness recovery in 50 unconscious postacute brain injury patients. EEG patterns were ranked according to Synek’s classification [85]. EEG was reactive in 48% of the patients, and 92% of the patients with reactive EEG recovered consciousness within 5 months of EEG recording. Furthermore, multivariable analysis indicated that an unconscious patient admitted to the rehabilitation unit within 2 months from brain injury, with a Level of Cognitive Functioning Scale score equal to 2 and the presence of reactive EEG, had a probability of recovery of consciousness higher than 97%. They concluded that EEG-R had a high predictive value for the prognosis of recovery of consciousness in the postacute phase of brain injury, with a high specificity (88.9%). In 2015, Bagnato et al. [50] analyzed EEG predictors of outcome in 106 patients with disorders of consciousness admitted for intensive rehabilitation and found that mean Coma Recovery Scale–Revised (CRS-R) scores were lower in patients without EEG-R than in patients with EEG-R, at admission and after 3 months. Moreover, patients without EEG-R had less CRS-R score improvement after 3 months than patients with EEG-R [50]. More recently, the same team reported that in a group of 28 patients with unresponsive wakefulness syndrome, 16 patients exhibited improved consciousness at 6 months [33]. EEG-R at admission was absent in all patients devoid of improved consciousness. Additionally, only patients with improved consciousness exhibited a reappearance of EEG-R after 6 months [33].

**Table 1:** Summary of findings regarding prognostic value of electroencephalographic reactivity in critically ill and postacute patients presenting with disorders of consciousness

Stimuli used for EEG reactivity testing	Study	Causes	Number of patients	Main reported prognostic value of EEG reactivity					Outcome times
				Main statements	Se %	Sp%	PPV%	NPP%	
Only nociceptive and/or tactile	Tsetsou et al. (2018) [81]	CA/H (TH)	61	EEG-R predicted good outcome	95 (75–99)	66 (49–80)	60 (42–76)	96 (79–99)	CPC at 3 months
	Rossetti et al. (2017) [73]	CA/H (TH)	357	Reactive EEG predicted good outcome with accuracy = 86.6% (82.6–90.0)			80.4 (75.9–84.4)		CPC at 3 months
	Topjian et al. (2016) [80]	CA/H (children)	128	Absence of reactivity was associated with worse EEG background category (*p* < 0.001), which is associated with death aOR = 3.63 (2.18–6.0) and unfavorable neurological outcome aOR = 4.38 (2.51–7.17).					PCPC at hospital discharge
	Li et al. (2015) [66]	Mixed	22	EEG-R to thermal stimulation(warm water 42 ± 2 °C) was elicited in 11 patients, and 9 of them showed improved outcomes. Among the 10 patients with no EEG-R, 9 patients did not improve.					mGOS at 1 year
	Lan et al. (2015) [64]	Mixed (children)	103	The poor-prognosis group had the lower proportion of events in reactive EEG patterns. Compared with patients with good prognosis, patients with poor prognosis had less frequent reactive EEG patterns as well as sleep architecture (*p* < 0.004).					Pediatric CPC
	Kang et al. (2014) [61]	Mixed	56	Performance of the variable reactive EEG for recovery of awareness: OR = 21.648 (2.212 to 211.870).	66.7 (44.7–83.6)	75.0 (56.2–87.9)	66.7 (44.7–83.6)	75.0 (56.2–87.9)	GOS at 1 year follow-up
Visual only	Bagnato et al. (2017) [33]	Mixed	28	5 of the 16 patients with consciousness improvement showed EEG-R on baseline EEG (at admission), which was absent in all patients without improvement.					CRS-R at 6 months
				Only patients with consciousness improvement showed the reappearance of EEG-R after 6 months. Nine of the 16 patients with consciousness improvement, corresponding to 81.9% of patients who did not show EEG-R at admission, had reappearance of EEG-R at the 6-month follow-up. On the contrary, none of the patients without consciousness improvement showed reappearance of EEG-R.					
	Nita et al. (2016) [38]	Mixed (children)	5	Intermittent photic stimulation induced reactivity of the burst-suppression pattern and standardized burst ratio reactivity appeared to reflect coma severity.					GCS
	Bagnato et al. (2015) [50]	Mixed	106	Mean CRS-R scores were lower for patients without EEG-R than for patients with EEG-R, at admission (5.4 ± 3.1 versus 10.7 ± 4.3) and after 3 months (10.6 ± 7 versus 21.2 ± 3.5).					CRS-R at 3 months
				Moreover, patients without EEG-R had less CRS-R score improvement after 3 months than patients with EEG-R (ANOVA, *F*_1,99_ = 21.5; *p* < 0.001).					
Auditory + nociceptive and/or tactile	Steinberg et al. (2018) [76]	Mixed	585	Reactive background EEG predicted survival aOR = 2.89 (1.49–5.59) and functionally favorable survival aOR = 1.51 (0.66–3.45).					CPC at hospital discharge
	Duez et al. (2018) [55]	Mixed	30	Nonreactive EEG predicted poor outcome	40 (23–68)	100 (69–100).			CPC at 3 months
	Johnsen et al. (2017) [37]	Neurosurgical	39	Nonreactive EEG predicted poor outcome	61 (42–77)	33 (06–76)	83 (62–95)	13 (2–42)	GOS at 3 months
	Azabou et al. (2016) [8]	CA/H	61	Nonreactive EEG predicted an unfavorable outcome with AUC 0.82.	84	80	98	31	GOS at 1 year
	Kang et al. (2015) [62]	Mixed	106	EEG-R predicted 1-month awakening from coma with AUC = 0.79 (0.71–0.88).	85.4 (71.6–93.5)	74.1 (60.7–84.4)	73.2 (59.5–83.8)	86.0 (72.6–93.7	CRS-R and CPC at 1 month
	Sivaraju et al., (2015) [75]	CA/H (TH)	100	Nonreactive EEG was associated with poor outcome	79 (66–88)	86 (66–95)	92 (81–98)	65 (47–79)	GOS at discharge
	Gilmore et al. (2015) [28]	Septic	98	Nonreactive EEG was associated with mortality					Mortality and mRS at 1 year
	Ribeiro et al. (2015) [71]	CA/H	36	Reactivity of the first EEG might predict better survival in post-cardiac arrest patients with hypoxic encephalopathy and generalized or bilateral lateralized periodic epileptiform discharges on first EEG (*p* = 0.0794).					Survival at hospital
	Su et al. (2013) [141]	CA/H (Stroke)	162	Dominant alpha wave without reactivity and dominant slow-wave rhythmic activity without reactivity were found to be correlated with poor outcome with ORs = 1.19 (0.27–5.14), and 1.82 (0.61–5.42), respectively.					mRS at 3 months
	Howard et al. (2012) [59]	CA/H	39	EEG-R to external stimuli (*p* = 0.039) and the presence of spontaneous fluctuations in the EEG (*p* = 0.003) were significantly associated with a favorable outcome.					mGOS at hospital discharge
	Zhang et al. (2011) [83]	CA/H (stroke)	161	Unfavorable EEG patterns, lack of EEG reactivity, pathologic N20 of SSEP, and pathological wave V of BAEP were associated with unfavorable outcome.	(92.4–97.0)	(82.5–99.5)			GOS at 6 months
	Logi et al. (2011) [14]	Mixed	50	EEG-R is a good prognostic factor of recovery of consciousness in the postacute phase of brain injury; nevertheless, its absence is not invariably associated with a poor prognosis.	68.7	88.9			LCFS at 5 months
				EEG reactivity predicted recovery of consciousness after 5 months from EEG recording with OR = 0.08 (0.01–0.44), *p* = 0.004 and 0.05 (0.01–0.53), *p* = 0.013, respectively, in univariable and multivariable logistic regression models.					
	Rossetti et al. (2010) [72]	CA/H	111	Unreactive EEG background was found in 3 of 45 (8%) survivors versus 53 of 65 (81%) nonsurvivors *p* = 0.001 (Fisher’s exact test). Unreactive EEG background was incompatible with good long-term neurological recovery (CPC 1–2) and was strongly associated with in-hospital mortality: aOR for death = 15.4 (3.3–71.9).					CPC at 3 and 6 months
	Gütling et al. (1995) [58]	severe TBI	50	All but one patient with preserved EEG reactivity (96%) had a good global outcome, but 93% of the patients in whom EEG reactivity was absent had a bad outcome. Using discriminant analysis, EEG-R correctly classified 92% of the patients into good or bad global outcome groups. EEG-R is an excellent long-term global outcome predictor, superior to the central conduction time of the somatosensory evoked potentials and GCS.					GOS at 1,5 years
Auditory + nociceptive and/or tactile + visual	Li et al. (2018) [65]	CA/H	73	EEG-R predicted survival with OR = 8.75 (1.48–51.95), *p* = 0.017.	82.1	84.1	86.8	78.7	GOS at 6 months
	Fernández-Torre et al. (2018) [57]	CA/H	26	In patients with a diagnosis of postanoxic alpha coma, theta coma, or alpha-theta coma, there was increased association of EEG-R with survival (*p* = 0.07).					CPC at 5 months
	Fantaneanu et al. (2016) [56]	CA/H (TH)	60	EEG-R varies depending on the stimulus modality as well as the temperature. EEG to nipple pressure is the most sensitive EEG-R test for outcome during hypothermia, with a good specificity, and is associated with good outcomes during either hypothermic or normothermic periods.	75	79.5			CPC at hospital discharge
	Braksick et al. (2016) [52]	Mixed	416	Absence of EEG-R was independently associated with in-hospital mortality:					In-hospital mortality
				OR = 8.14 (4.20–15.79)					
	Mohammad et al. (2016) [68]	Septic (children)	119	A nonreactive background was noted in 48% (57 of 119) of patients on their first EEG and predicted abnormal outcome in children with encephalitis (OR = 3.8, *p* < 0.001).					LOS at last follow-up
	Juan et al. (2015) [60]	CA/H	197	Seventy-two patients (37%) had a nonreactive EEG background during TH, with 13 (18%) evolving toward reactivity in NT. Compared with those remaining nonreactive (*n* = 59), they showed significantly better recovery of brainstem reflexes (*p* < 0.001), better motor responses (*p* < 0.001), transitory consciousness improvement (*p* = 0.008), and a tendency toward lower NSE (*p* = 0.067).					CPC at 3 months
	Oddo and Rossetti (2014) [69]	CA/H (TH)	134	AUC for nonreactive hypothermic EEG for predicting mortality and poor outcome were 0.86 (0.81–0.92) and 0.81 (0.75–0.87), respectively					CPC at 3 months
	Crepeau et al. (2013) [54]	CA/H	54	Nonreactive EEG was associated with poor outcome with OR = 17.05 (3.22–90.28).					CPC at hospital discharge
	Sutter et al. (2013) [78]	Mixed	105	Nonreactive EEG background was independently associated with death in encephalopathic patients with triphasic waves: OR = 3.73 (1.08–12.80, *p* = 0.037).					Mortality and CPC at discharge
	Bisschops et al. (2011) [51]	CA/H (TH)	103	EEG was unreactive in 15 of 23 patients (65.2%) with an unfavorable outcome and in none of the 4 patients with a good outcome (*p* = 0.015).			100 (75–100)		GOS at hospital discharge
	Rossetti et al. (2010) [74]	CA/H (TH)	34	Nonreactive cEEG background during therapeutic hypothermia had false-positive rate of 0 (0–18%) for mortality. All survivors had cEEG background reactivity, and the majority of them (14 [74%] of 19) had a favorable outcome.			100% (74 to 100%)		CPC at 2 months
	Ramachandrannair et al. 2005 [70]	Mixed (children)	33	Among the 19 children with nonreactive EEG, 13 (65%) had unfavorable outcomes, including 10 deaths. Outcome was better in children with EEG-R (*p* = 0.023). EEG-R was associated with a lower PCOPCS score at follow-up (*p* = 0.002).					PCOPCS at 1 year
	Amantini et al. (2005) [49]	Severe TBI	60	Awakening prediction with EEG-R: LR+ = 1.6 (0.8–3.2).	66.7	60.0	83.3	37.5	GOS at 1 year
				Good outcome prediction with EEG-R: LR+ = 1.8 (1.2–2.9).	79.3	58.1	63.9	75.0	
	Young et al. (1999) [82]	Mixed	214	Nonreactive EEG was one of the individual factors strongly related to mortality: OR > 2.0.			> 0.80		
				EEG-R was among factors that favored survival rather than death.					
	Kaplan et al. (1999) [44]	Mixed	36	Presence of EEG reactivity in alpha coma correlated with survival (χ^2^ = 5.231; *p* = 0.022). If the EEG showed no reactivity after cardiac arrest, patients were likely to die (χ^2^ = 3.927; *p* = 0.0475).					GOS after hospital discharge
Not described	Søholm et al. (2014) [30]	CA/H	219	A favorable EEG pattern (including reactivity) was independently associated with reduced mortality with HR 0.43 (0.24–0.76), *p* = 0.004 (false-positive rate, 31%) and a nonfavorable EEG pattern (including no reactivity) was associated with higher mortality (HR = 1.62, 1.09–2.41, *p* = 0.02) after adjustment for known prognostic factors (false-positive rate, 9%).					30-day mortality and CPC at hospital discharge
	Kessler et al. (2011) [63]	CA/H (TH) Children	35	During hypothermia, patients with EEGs in categories 2 (continuous but unreactive EEG) or 3 (discontinuity, burst suppression, or lack of cerebral activity) were far more likely to have poor outcome than those in category 1 (continuous and reactive EEG) (OR = 10.7, *p* = 0.023, and OR = 35, *p* = 0.004, respectively). Similarly, for EEG obtained during normothermia, patients with EEGs in categories 2 or 3 were far more likely to have poor outcomes than those in category 1 (OR = 27, *p* = 0.006, and OR = 18, *p* = 0.02, respectively).					PCPC at hospital discharge
	Thenayan et al. (2010) [79]	CA/H	29	Of the 18 patients with nonreactive EEG, only 1 recovered awareness; of the 11 patients with EEG-R, 10 recovered awareness.	90 (57–100)	94 (70–100)			Awakening during hospitalization
	Claassen et al. 7(2006) [53]	SAH	116	Outcome was poor in all patients with absent EEG reactivity					3-month mRS

*Abbreviations*: *ANOVA* Analysis of variance, *BAEP* Brainstem auditory evoked potential, *Se* Sensitivity, *Sp* Specificity, *PPV* Positive predictive value, *NPV* Negative predictive value, *aOR* Adjusted OR, *CA/H* Cerebral anoxia/hypoxia, *TH* Target therapeutic hypothermia, *NT* Normothermia, Mixed = Heterogeneous population of critically ill or postacute patients with disorders of consciousness from various causes (toxic, septic, metabolic, or vascular). *CPC* Cerebral Performance Categories scale, *PCPC* Pediatric Cerebral Performance Category scale, *PCOPCS* Pediatric Cerebral and Overall Performance Category scale, *GCS* Glasgow Coma Scale, *GOS* Glasgow Outcome Scale, *mGOS* Modified Glasgow Outcome Scale, *mRS* Modified Rankin Scale, *LCFS* Level of Cognitive Functioning Scale, *LOS* Liverpool Outcome Score, *CRS-R* Coma Recovery Scale–Revised, *cEEG* Continuous electroencephalography, *SAH* Subarachnoid hemorrhage, *NSE* Neuron-specific enolase, *LR+* Positive likelihood ratio

In 1999, Kaplan et al. performed a retrospective analysis of the value of EEG-R to noxious stimuli for predicting outcome in 36 cases of alpha coma patients [44]. Fourteen of the 19 patients with nonreactive EEG died; 2 had support discontinued; and only 3 awoke. Kaplan et al. concluded that, although the cause of alpha coma largely predicted outcome, EEG-R predicted survival because most patients with EEG-R awoke, whereas most of those without EEG-R died [44]. Fernández-Torre et al. showed that in 26 patients with a diagnosis of postanoxic alpha coma, theta coma, or alpha-theta coma, EEG-R was associated with survival (*p* = 0.07) [57]. In 2009, Rossetti et al. found that postanoxic status epilepticus patients with favorable outcome exhibited preserved brainstem reflexes, cortical somatosensory evoked potentials (SSEPs), and reactive EEG background [18]. The same team demonstrated in 2010 that EEG background reactivity was useful in determining a prognosis in cardiac arrest survivors treated by therapeutic hypothermia [72]. In addition, median serum neuron-specific enolase peak values were higher in patients with nonreactive EEG background and discontinuous patterns, suggesting increased neuronal damage, and all subjects with nonreactive EEGs died [16]. Of the 36 patients studied by Ribeiro et al. [8], who had postanoxic encephalopathy showing generalized periodic epileptiform discharges on their first EEG, clinical characteristics between survivors and nonsurvivors did not significantly differ except for a trend toward significance for the presence of reactivity on the first EEG [71]. In our recent prospective study of 61 postanoxic patients with coma, the EEG was nonreactive in 48 patients, of whom 46 (95.8%) had an unfavorable outcome, defined as death, vegetative state, minimal conscious state, or severe disability [8]. We found that nonreactive EEG had a high sensitivity and specificity similar to those of the well-established Synek score for predicting an unfavorable outcome [3, 14, 15, 22, 84, 86, 87]. In accordance with Gilmore et al. [28], who showed that a lack of EEG-R was associated with mortality up to 1 year following discharge in ICU patients with sepsis, we recently found in a population of 110 patients with sepsis that ICU mortality was independently associated with the absence of EEG-R [27]. Furthermore, absence of EEG-R correlated with later development of in-ICU delirium. The absence of EEG-R and subsequent occurrence of delirium might be related to an impairment of cortical or brainstem function [88]. A possible role of sedation in the abolition of EEG-R may be hypothesized because administration of midazolam has been shown to increase the risk of delirium [89]. However, absence of EEG-R did not correlate with midazolam infusion rates or with the Richmond Agitation-Sedation Scale score in our study. Conversely, unfavorable outcomes in patients who nevertheless present EEG responsiveness is also observed [14, 62]. This may be related to a lack of standardization of stimulations as previously discussed. Unfortunately, the procedure is rarely detailed in the literature.

The exact protocols and types of stimuli used for assessing EEG-R are quite heterogeneous, but three modalities of stimuli are used: the somesthetic modality, the auditory modality, and visual modality. Among the 42 studies in the present review, the 3 modalities were jointly tested in 15 (36%); both the somesthetic and auditory modalities were jointly tested in 14 (33%); 6 (14%) studies used only the somesthetic modality; and 3 (7%) studies used the visual modality alone. Stimulation modality was not described in four studies (10%). The visual modality is less frequently used, probably because the visual pathways are a little more difficult to assess in comatose patients compared with the auditory and somesthetic pathways. Johnsen et al. [37], systematically using all three stimulation modalities for EEG-R assessment, demonstrated that the nociceptive modality was the most effective type of stimulation (20.4%), followed by the auditory (8.7%) and visual (6.7%) modalities. Discrimination between good and poor outcomes was best in the theta and alpha bands for nociceptive stimulation in the first 10–20 seconds and for auditory stimulation in the first 5–10 seconds, whereas eye opening did not discriminate between good and poor outcomes [37]. This differential sensitivity between types of stimulation might be explained by high levels of noise and light in the ICU environment, rendering these two stimulation modalities less sensitive than nociceptive stimulation. However, Nita et al. demonstrated in a small group of five comatose children with acquired brain injury of various etiologies that intermittent photic stimulation performed at 1 Hz for 1 minute induced reactivity of the burst-suppression pattern and that standardized burst ratio reactivity appeared to reflect coma severity [38].

## Discussion

**Fig. 2:**
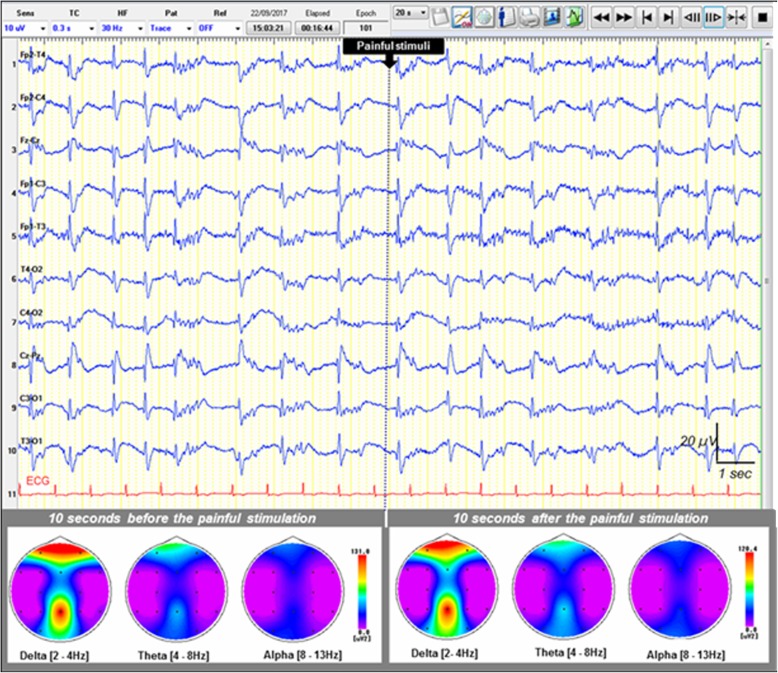
Example of a nonreactive electroencephalogram (EEG) following painful stimulus (pinching) in a patient with impaired consciousness. Upper: A 20-second epoch EEG sample showing generalized pseudoperiodic discharges of spikes with no change after the painful stimulus (pinching) in a postanoxic ICU patient. Recording: 20 mm/s, sensitivity: 10 μV/mm; filter settings: 0.50–70 Hz. Lower: EEG spectral power featuring topographic mapping of power of each main EEG frequency band (delta, theta, and alpha) computed 10 seconds before and 10 seconds after the painful stimulus. No significant EEG frequency band power change was observed after the painful stimulus. Higher-power values are shown in warm colors, and cool colors depict lower power

**Fig. 3:**
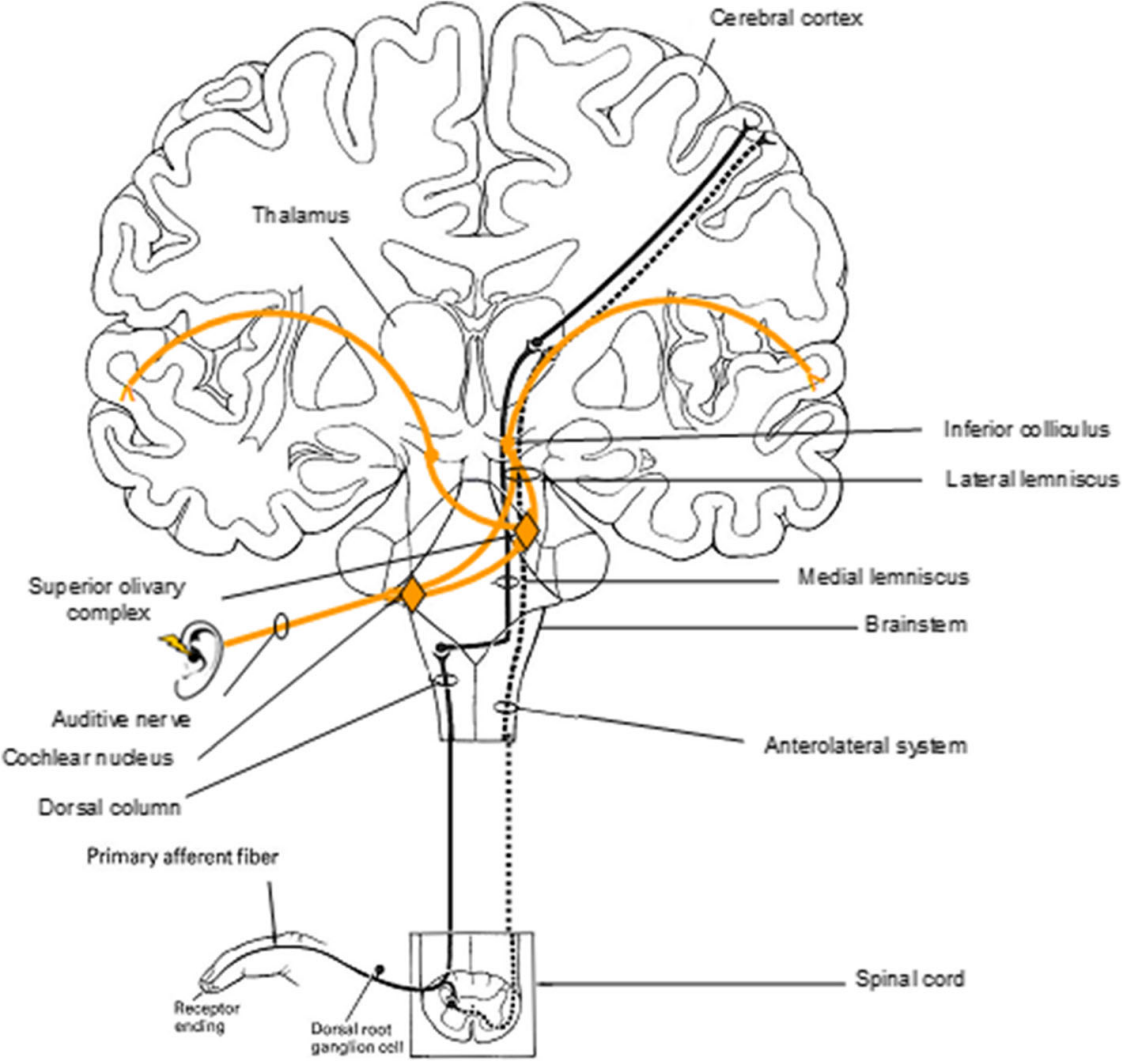
Schematic representation of pathways that convey somatosensory and auditory information to the cerebral cortex. The dorsal column-medial lemniscus system (*solid black line*), anterolateral-extralemniscal system (*broken line*), and auditory-lateral lemniscal system (*orange colored solid line*) are shown

Diffuse neurological failure, usually manifesting as coma and delirium, is a major determinant of mortality and morbidity in the ICU [90]. Lack of EEG-R correlated with mortality in patients with impaired consciousness [14, 16, 18, 72, 84]. Although there is no consensus regarding standardized methodology, EEG-R in patients with impaired consciousness is conventionally assessed through the application of two external stimuli: auditory and/or nociceptive stimulation [31], as well as, more rarely, passive eye opening and intermittent photic stimulation, both in adults [31, 33, 50] and in children [38]. The EEG is considered reactive when one of these stimulations modifies the amplitude and/or frequency of the background activity (Fig. 1) [1, 5–7]. Nonreactive EEG is characterized by no change in cerebral EEG activity after auditory and painful stimuli. Figure 2 features a nonreactive EEG following nociceptive stimulation in a postanoxic patient. EEG-R to auditory or painful stimuli can be seen as the modulation of the cortical activity following a peripherally applied stimulation. EEG-R to auditory stimuli requires the functional integrity of the peripheral and central auditory pathways involving the inner ear, the bulbopontine junction, the middle and upper parts of the pons, the midbrain (lateral lemniscus), the inferior colliculus, the medial geniculate nucleus of the thalamus, and the primary auditory cortex [91], whereas EEG-R to painful stimuli requires functional integrity of the pain projection pathways [92, 93] and the anterolateral system (Fig. 3) [94]. EEG-R to intense nociceptive and auditory stimuli indirectly tests the proper functioning of the somatosensory and auditory pathways of the brainstem and the cerebral cortex through two complementary modalities. In cases of severe cerebral impairment, the afferent nociceptive sensory or auditory impulses generated by the peripheral stimuli cannot reach the cerebral cortex, and EEG is therefore nonreactive. Critically ill patients are at risk of brain dysfunction induced not only by primary brain insults but also by neuroinflammation [95], ischemia secondary to microcirculatory dysfunction, and the neurotoxic effect of metabolic disturbance leading to impaired membrane excitability, neural conduction, and neural loss [96–98]. Impaired central auditory [99–102] and somatosensory [103–105] pathways have been documented by studies of evoked potentials to be associated with increased mortality in patients with impaired consciousness. Studies investigating the prognostic value of laser-evoked potentials and their correlation with EEG-R may be useful [106]. However, measuring laser-evoked potentials in the ICU is time-consuming compared with EEG. The brainstem controls many vital functions, including cardiocirculatory, respiratory, and arousal, through the ascending reticular activating system. Ascending monoaminergic and cholinergic activating systems localized in the upper brainstem, posterior hypothalamus, and basal forebrain release neurotransmitters, such as acetylcholine, norepinephrine, serotonin, histamine, and glutamate, and innervate the cerebral cortex, thalamus. They therefore have a widespread influence on forebrain function [107]. The brainstem also houses the autonomic nervous system’s main centers, which modulate immunity and systemic immune responses to aggression [108, 109]. Impaired EEG-R could therefore at least partly reflect a brainstem dysfunction in patients with impaired consciousness [110, 111]. EEG-R to visual stimulation (passive eye opening and intermittent photic stimulation) requires a functional integrity of the visual pathways from the retina to the occipital visual cortex, including the optic nerve, optic chiasm, optic tract, lateral geniculate nucleus, optic radiations, and striate cortex. A loss of EEG-R may reflect extensive damage to cortical or subcortical structures. Animal experiments have demonstrated that EEG-R is associated with the structural and functional integrity of the corticothalamic loop and thalamus-brainstem loop [112]. The thalamus is the key relay structure for ascending peripheral sensorial inputs (somesthetic, auditory, or visual) toward the cerebral cortex. The thalamus and its recurrent connections with the cortex play an integral role in the generation and sustenance of brain rhythms that underlie brain function as measured by EEG [113, 114]. The reticular nucleus of the thalamus (RN) surrounds the rostral and lateral surfaces of the dorsal thalamus. The RN contains exclusively GABAergic neurons and, via extensive inhibitory outputs, modulates all incoming sensory information on its way to the cerebral cortex [115]. The RN therefore plays a critical role in controlling the firing patterns of ventroposterior thalamic neurons and is thought to play a critical role in controlling thalamocortical rhythm [116]. The RN plays a crucial role in selective attention and consciousness because it can inhibit the area of the thalamus from which the initial information came and can influence the flow of information between the thalamus and cortex [117]. Increases in low-frequency cortical power may be due to a shift in thalamic neuron activity from a state dominated by tonic firing to one in which there is an increase in low-threshold spike burst firing [118]. Low-threshold calcium bursts occur when thalamocortical relay cells are in a state of hyperpolarization; there is evidence that the RN is capable of entertaining this “burst-firing mode” [119], and it is argued that the RN serves to maintain the low-frequency thalamocortical oscillations (4–10 Hz) [120, 121]. Aberrations and alterations in these thalamocortical loops is characteristic of several central nervous system disorders, particularly disorders of consciousness [122], because human perceptions arise from ongoing activity within recurrent thalamocortical circuits [123]. The lack of EEG-R observed in critically ill patients may result from altered modulation of thalamocortical loop activity by the afferent sensorial input due to the neural impairment [118]. This unresponsiveness of the thalamocortical rhythm’s synchronization or desynchronization [107, 113, 124] to sensorial stimuli reveals cerebral impairment and is strongly associated with patient outcome [14, 16, 18, 72, 84]. Moreover, the same EEG pattern may have a different prognostic value, depending on the presence or lack of EEG-R [44, 46, 125].

Most studies of EEG-R do not mention the exact time at which reactivity was evaluated; however, it is well known that EEG features may change during the acute stage, especially in the first 24–48 hours after cardiac arrest [75, 126, 127]. The impact of the recovery of EEG-R on patient prognosis was recently demonstrated by Bagnato et al. [33], who reported that only patients with consciousness improvement showed the reappearance of EEG-R. Nine of the 16 patients with consciousness improvement, corresponding to 81.9% of patients who did not show EEG-R at admission, had reappearance of EEG-R at the 6-month follow-up. On the contrary, none of the patients without consciousness improvement showed reappearance of EEG-R. Repeated standard EEG or continuous EEG monitoring is then recommended in order to closely follow trends of the EEG changes in acute patients [27, 128–130].

It should be mentioned that EEG background activity and SSEPs are other neurophysiological parameters with robust outcome-predictive values in patients with impaired consciousness [1, 128, 131]. EEG background activity reflects spontaneous global cerebral functioning. It usually worsens by slowing down, decreasing amplitude, flattening, and discontinuing according to the severity of brain dysfunction [1, 5]. Worsened EEG background activity has been associated with unfavorable outcome in several studies [26, 75, 85, 130, 132]. Reduced EEG amplitudes and delta frequencies correlated with worse clinical outcomes, whereas alpha frequencies and reactivity correlated with better outcomes in patients with disorders of consciousness admitted for intensive rehabilitation [50]. Low-voltage or flat EEG background activity, burst suppression, and burst suppression with identical bursts are constantly associated with unfavorable outcome in postanoxic coma patients [75, 130, 132]. Spontaneously discontinuous background predicted unfavorable outcome with a false-positive rate of about 7% (95% CI, 0–24%) [16], whereas a continuous background predicted awakening with positive predictive values of 92% (95% CI, 80–98%) [133] and 72% (95% CI, 55–88%) [75]. SSEPs explore the functional integrity of the somatosensory pathways from the peripheral level to the cortical one through the brainstem and subcortical levels. The ability of absent SSEPs to detect patients at risk for poor neurological outcome appears to be robust [134]. Bilateral absent cortical components of SSEPs were associated with no awakening in anoxic coma, but normal SSEPs had less predictive capacity in the same cohort [135] because only 52% of patients with normal SSEPs awoke from coma [135]. In patients with TBI, normal SSEPs after TBI are associated with a 57% chance of good recovery, whereas bilateral absent SSEPs are associated with only a 1% chance of functional recovery [135, 136]. When combined with absent EEG-R, the prognostic value of SSEPs further increased [137]. Although there is no systematic study comparing the prognostic value of EEG background activity, SSEP, and EEG-R, available data and guidelines suggest that a combined multimodal assessment with these tests increases the accuracy of outcome prediction in patients with impaired consciousness [5, 128, 138–140].

## Conclusions

This review emphasizes that whatever the etiology, patients with impaired consciousness featuring a reactive EEG are more likely to have favorable outcomes, whereas those with a nonreactive EEG are prone to unfavorable outcome. EEG-R is, then, a valuable prognostic parameter and warrants a rigorous assessment. However, current assessment methods are heterogeneous, and no consensus exists. Standardization of stimulation and interpretation methods is needed. Furthermore, it should be stated that all other EEG basic parameters, such as the dominant frequency or the continuity, warrant assessment in order to provide a fully integrated interpretation.

## Abbreviations

ANOVA: Analysis of variance
aOR: Adjusted OR
BAEP: Brainstem auditory evoked potential
CA/H: Cerebral anoxia/hypoxia
cEEG: Continuous electroencephalography
CPC: Cerebral Performance Categories scale
CRS-R: Coma Recovery Scale–Revised
EEG: Electroencephalography, electroencephalogram
EEG-R: Electroencephalographic reactivity
GCS: Glasgow Coma Scale
GOS: Glasgow Outcome Scale
ICU: Intensive care unit
LCFS: Level of Cognitive Functioning Scale
LOS: Liverpool Outcome Score
LR+: Positive likelihood ratio
mGOS: Modified Glasgow Outcome Scale
mRS: Modified Rankin Scale
NPV: Negative predictive value
NSE: Neuron-specific enolase
NT: Normothermia
PCOPCS: Pediatric Cerebral and Overall Performance Category scale
PCPC: Pediatric Cerebral Performance Category scale
PPV: Positive predictive value
RN: Reticular nucleus of the thalamus
SAH: Subarachnoid hemorrhage
Se: Sensitivity
Sp: Specificity
SSEP: Somatosensory evoked potential
TBI: Traumatic brain injury
TH: Target therapeutic hypothermia
